# HDL-C as a novel predictor of immune reconstitution in people living with HIV: insights from a baseline-to-dynamic change cohort study in China, 2005–2022

**DOI:** 10.3389/fimmu.2025.1520615

**Published:** 2025-05-12

**Authors:** Xiaorui Li, Liqin Sun, Yun He, Fang Zhao, Yinsong Luo, Chenye Liu, Yiyao Hu, Yuxin Jiang, Hongzhou Lu, Jiaye Liu

**Affiliations:** ^1^ School of Public Health, Shenzhen University Medical School, Shenzhen, China; ^2^ Department of Infectious Diseases, National Clinical Research Center for Infectious Diseases, Shenzhen Third People’s Hospital, Shenzhen, China

**Keywords:** high-density lipoprotein cholesterol, HIV, Cd4 + t cell, trajectory, immune reconstitution

## Abstract

**Background:**

High-density lipoprotein cholesterol (HDL-C) is a well-established marker of lipid metabolism and increasingly recognized as an indicator of inflammatory status. This study investigates HDL-C’s role as a predictor of immune reconstitution in people living with HIV (PLWH).

**Methods:**

We performed a prospective cohort study of 15,434 PLWH initiating antiretroviral therapy at the Third People’s Hospital of Shenzhen, China, between 2005 and 2022. Baseline quartile grouping and Group-Based Trajectory Modeling (GBTM) explored the relationship between HDL-C and immune reconstitution. Restricted cubic spline plots identified nonlinear association.

**Results:**

Over a median follow-up of 17.9 months, 9,609 PLWH achieved the CD4+ T-cell count of 500 cells/μL or higher. Multivariate-adjusted Cox proportional hazards regression model showed that hazard ratios (HR) [95% confidence intervals (CI)] for CD4+ T-cell recovery in Q2, Q3, and Q4 versus Q1 of the HDL-C were 0.94 (95% CI: 0.88-0.99), 0.92 (95% CI: 0.87-0.98), and 0.85 (95% CI: 0.80-0.91), respectively. GBTM identified two HDL-C trajectories: Low-floating and High-floating. Relative to Low-floating, High-floating demonstrated a reduced likelihood of CD4+ T-cell recovery (HR=0.86, 95% CI: 0.82-0.90, p < 0.001). A nonlinear association was observed between HDL-C and the outcome (p for nonlinear association = 0.037, p for overall < 0.001), with a threshold at 1.13 mmol/L. Negative correlations between HDL-C and CD4+ T-cell recovery were observed both below the threshold (HR=0.72, 95% CI: 0.57-0.92) and above the threshold (HR=0.78, 95% CI: 0.69-0.87) (both p < 0.05).

**Conclusions:**

Our study highlights HDL-C’s role in immune recovery, suggesting its potential in guiding prevention and treatment strategies for PLWH.

## 1 Introduction

Globally, there were 1.3 million new HIV infections in 2022, and 29.8 million of the 39 million people living with HIV (PLWH) were receiving antiretroviral therapy (ART) by 2023 (1). While ART has significantly improved the survival and quality of life for PLWH, a subset of patients continues to experience inadequate immune recovery, as indicated by CD4+ T lymphocyte (CD4+ T-cell) counts that fail to return to the normal range (2, 3). This incomplete immune restoration contributes to persistently elevated rates of both HIV-related and non-HIV-related morbidity and mortality. Thus, the identification of reliable and valid predictive markers is essential for developing strategies to improve the long-term health outcomes of PLWH.

Despite extensive research offering valuable insights into factors influencing CD4+ T-cell recovery in PLWH, these factors remain insufficient to fully capture the complex and dynamic nature of immune recovery (4–6). Baseline CD4+ T-cell count, ART regimen, patient age and gender, and HIV RNA levels provide useful information but fail to encompass the intricate processes of immune reconstitution, long-term inflammation, and the multidimensionality nature of HIV pathogenesis (7–12). For example, PLWH with similar baseline characteristics may exhibit markedly different recovery trajectories. This limitation underscores the need for more robust and predictive markers that can better capture the evolving immune landscape in PLWH.

Existing research indicates that lipid metabolism plays a multifaceted role in the immune system, with high-density lipoprotein cholesterol (HDL-C) emerging as a potentially important marker in relation to immune function and disease progression (13, 14). Traditionally, HDL-C has been recognized as a protective factor for cardiovascular health (15). However, emerging research increasingly suggests that HDL-C plays a significant role in immune regulation (16–19). Studies have reported that low HDL-C levels are associated with an increased risk of autoimmune diseases, and HDL-C levels exhibit a U-shaped relationship with the risk of mortality from infectious diseases (20, 21). Given the chronic inflammation and immune dysregulation associated with HIV infection, it is plausible that HDL-C may play a significant role in immune reconstitution. Nevertheless, the potential influence of HDL-C on CD4+ T-cell recovery in PLWH remains inadequately explored.

In light of these considerations, we undertook a study of both baseline and dynamic HDL-C levels to investigate the predictive value of HDL-C in immune reconstitution among PLWH.

## 2 Methods

### 2.1 Study design and participants

We conducted a prospective cohort study of treatment-naive PLWH who sought care at the Third People’s Hospital of Shenzhen, China, between 2005 and 2022. This hospital is the sole designated facility for HIV treatment and management in Shenzhen. The following patients were excluded from the study: (1) those with a CD4+ T-cell count exceeding 500 cells/µL (n = 1315) prior to the initiation of ART, (2) those without any follow-up records (n=604), and (3) those with HDL-C outliers (n=432). Ultimately, a total of 15,434 individuals were included in the final analysis.

We collected baseline and follow-up data, including socio-demographic characteristics, clinical information (exposures and outcomes), and laboratory test results obtained from survey questionnaires and the hospital’s routine diagnosis and treatment information systems. Patients were followed up every three months.

The study protocol adhered to the ethical guidelines of the 1975 Declaration of Helsinki and was approved by the Institutional Review Board of Shenzhen Third People’s Hospital (No. 2022-143). All participants provided written informed consent.

### 2.2 Assessment of covariates

Our study included the following categories of covariates obtained from the hospital records: (1) demographic characteristics included age, sex, marital status (married or cohabiting, divorced or separated, widowed, never married), body mass index (BMI), which is calculated by dividing weight in kilograms by height in meters squared, smoking status (ever vs. never), and alcohol consumption (yes or no); (2) laboratory parameters included glucose, low-density lipoprotein cholesterol (LDL-C), triglycerides (TG), total cholesterol (TC), creatinine, white blood cells (WBC), and platelet count; (3) comorbidities included hepatitis B virus (HBV) infection (positive HBV surface-antigen test or positive envelope-antigen test, or detectable HBV DNA), and hepatitis C virus (HCV) infection (positive anti-HCV antibody test or detectable HCV RNA); (4) HIV-related factors included the route of HIV transmission (male-to-male sexual contact, heterosexual contact, injection drug use, or other), the CD4+ T-cell count and CD8+ T-cell count at ART initiation, HIV RNA levels, presence of opportunistic infection (yes, no), and the time interval between HIV diagnosis and ART initiation. The HDL-C levels were obtained through standardized hospital laboratory tests.

### 2.3 Follow-up and outcomes

The primary outcome of this study was CD4+ T-cell recovery to 500 cells/μL or higher within five years. Baseline was defined as the date PLWH first received ART at the participating hospital. Outcomes were assessed from three months after cohort entry until death, loss to follow-up, or Dec 31, 2023, the final date of follow-up, whichever occurred first. PLWH were considered lost to follow-up if they missed scheduled appointments for more than nine months, according to standard clinical practice.

### 2.4 Statistical analysis

All statistical analyses were performed using R software (version 4.4.0; https://www.r-project.org) and SAS software (version 9.4). Participants were categorized into four groups according to HDL-C quartiles. Continuous variables were presented as mean and standard deviation (SD), while categorical variables were summarized as frequency and percentage. Statistical methods included one-way ANOVA for continuous variables and the chi-square test for categorical variables.

We applied the group-based trajectory modeling (GBTM) approach, implemented through SAS Proc Traj, to identify subgroups within each group that exhibited a similar underlying trajectory of HDL-C (22, 23). GBTM an application of finite mixture modeling, is used to identify groups of individuals following comparable trajectories of variables over time (24, 25). Before fitting the model, likelihood ratio testing was conducted to determine the best-fit polynomial shape for each vital sign (i.e., linear, quadratic, cubic) (26). To select the optimal GBTM model, we compared models with two to five trajectory groups based on the following criteria:(1) the Bayesian Information Criterion (BIC), with lower values indicating better model fit; (2) the average posterior probabilities (Avepp) of group membership, with values greater than 0.7 considered acceptable; and (3) sufficient patient representation, defined as a proportion greater than 5% in each group (27, 28). As shown in **Supplementary Table S1**, the model with two trajectory groups and cubic polynomials terms for each trajectory (trajectory-group 2) demonstrated the best balance of model fit (BIC = -8552.88), classification accuracy (Avepp > 0.97 for all groups), and adequate representation across groups. Therefore, the trajectory-group 2 was selected as the final model.

Kaplan-Meier cumulative incidence plots were used to evaluate the relationship between HDL-C quartiles and CD4+ T-cell counts reaching at least 500 cells/μL during the follow-up period. Simultaneously, these plots also analyzed the association between different HDL-C trajectory groups and CD4+ T-cell counts exceeding 500 cells/μL throughout the follow-up. A log-rank test was employed for statistical evaluation. We developed a multivariate Cox proportional hazards model to examine the correlation between HDL-C levels and achieving a CD4+ T-cell count of 500 cells/μL or more. We constructed three models to adjust for potential confounders in CD4+ T-cell recovery: Model 1 was unadjusted, serving as a baseline; Model 2 was adjusted for age and sex to account for basic demographic influences on CD4+ T-cell recovery; Model 3 included further adjustments for age, sex, BMI, smoking, alcohol consumption, CD4+ T-cell count, CD8+ T-cell count, CD4/CD8 ratio, white blood cells, HIV RNA, HBV, HCV, opportunistic infections, the time interval between HIV diagnosis and ART initiation, HIV transmission route, and ART regimen. These variables were selected based on their established or potential roles as confounders or modifiers in CD4+ T-cell recovery process. The increase in predictive risk after adding HDL-C to the conventional risk model was assessed through changes in Net Reclassification Improvement (NRI) and Integrated Discrimination Improvement (IDI).

To explore and further investigate the relationship between HDL-C and CD4+ T-cell recovery, we utilized a restricted cubic spline plot to assess the potential nonlinearity of this relationship. After identifying a nonlinear relationship, we applied a two-piecewise multivariate Cox proportional hazards model to analyze both segments, divided at the HDL-C inflection point. The threshold was estimated using the maximum likelihood method. Subsequently, stratification and interaction analyses were performed by age, sex, BMI, marital status, HIV transmission route, smoking, alcohol consumption, HBV, HCV, HIV RNA, CD4+ T-cell count, CD8+ T-cell count, CD4/CD8 ratio, opportunistic infection, the time interval between HIV diagnosis and ART initiation and ART regimen. In sensitivity analyses, we excluded individuals within the six months of follow-up, irrespective of whether they had achieved a CD4+ T-cell count 500 cells/μL or more, and conducted another analysis that restricted the sample to participants enrolled within the past 10 years.

Since our data were derived from hospital electronic medical records, not all covariate data were complete. We used the random forest model for multiple imputation of missing covariates when the missing proportion was below 1% to obtain unbiased estimates of the relationship between HDL-C levels and CD4+ T-cell recovery. Statistical significance was defined as a p-value of less than 0.05.

## 3 Results

### 3.1 Baseline characteristics of participants

Among the 15,434 participants included in the study, 13,896 (90.0%) were male. The mean age of the participants was 34.5 years, with a SD of 10.6 years. As shown in **Table 1**, participants’ baseline characteristics were stratified by HDL-C quartiles: Q1 (0.21-0.98 mmol/L), Q2 (0.99-1.17 mmol/L), Q3 (1.18-1.37 mmol/L), and Q4 (1.38-3.48 mmol/L). Compared to those in the lowest quartile (Q1), participants in the higher HDL-C quartiles were generally younger, more likely to be female, had a lower BMI, and were more likely to consume alcohol. They also exhibited higher levels of TC and creatinine, and lower platelets counts. Additionally, they had lower proportions of HIV RNA > 100,000 copies/mL and higher proportions of HBV infection. Conversely, they showed lower CD4+ T-cell counts and higher CD8 counts. We tested for the optimal number of HDL-C trajectories to explain the heterogeneity in this population. The best-fit GBTM model identified two distinct trajectories, labelled as Low-floating (n = 10,854), High-floating (n = 4,580) (**Supplementary Figure S1**, **Supplementary Table S1**). Furthermore, we observed that HDL-C trajectory groups showed significant differences in multiple baseline characteristics, consistent with trends seen in HDL-C quartiles. In these models, the Low-floating group exhibited relatively low HDL-C levels, with a median similar to Q1, while the High-floating group was closer to Q4 (**Supplementary Figure S1**). Participants in the High-floating group had higher glucose levels and lower LDL-C levels compared to those in the Low-floating group. Additionally, the time interval between HIV diagnosis and ART initiation was shorter in participants with High-floating (all p < 0.05). Other characteristics were similar across the HDL-C trajectory groups. Similar trends were also observed in the three-group and four-group trajectory analyses (**Supplementary Tables S2** and **S3**).

**Table 1:** Baseline characteristics according to the HDL-C quartiles and Trajectory−Group.

Characteristics^a^	Quartiles of HDL-C						Trajectory−2Groups of HDL-C		
	Total	Q1(0.21-0.98)	Q2(0.99-1.17)	Q3(1.18-1.37)	Q4(1.38-3.48)	*P*-value	Low-floating	High-floating	*P*-value
N	15434	3983	3771	3875	3805		10854	4580	
Sex						<0.001			<0.001
male	13896 (90.0)	3770 (94.7)	3548 (94.1)	3512 (90.6)	3066 (80.6)		10273 (94.6)	3623 (79.1)	
female	1538 (10.0)	213 (5.3)	223 (5.9)	363 (9.4)	739 (19.4)		581 (5.4)	957 (20.9)	
Age, years						<0.001			<0.001
18-24	2553 (16.5)	562 (14.1)	651 (17.3)	693 (17.9)	647 (17.0)		1808 (16.7)	745 (16.3)	
25-34	6901 (44.7)	1732 (43.5)	1704 (45.2)	1774 (45.8)	1691 (44.4)		4941 (45.5)	1960 (42.8)	
35-44	3561 (23.1)	981 (24.6)	871 (23.1)	830 (21.4)	879 (23.1)		2496 (23.0)	1065 (23.3)	
>=45	2419 (15.7)	708 (17.8)	545 (14.5)	578 (14.9)	588 (15.5)		1609 (14.8)	810 (17.7)	
BMI, kg/m^2^						<0.001			<0.001
<18.5	2464 (16.0)	583 (14.6)	541 (14.3)	611 (15.8)	729 (19.2)		1495 (13.8)	969 (21.2)	
18.5-23.9	10174 (65.9)	2453 (61.6)	2507 (66.5)	2618 (67.6)	2596 (68.2)		7069 (65.1)	3105 (67.8)	
>=24	2796 (18.1)	947 (23.8)	723 (19.2)	646 (16.7)	480 (12.6)		2290 (21.1)	506 (11.0)	
Marital status						<0.001			<0.001
Never married	9173 (59.4)	2401 (60.3)	2327 (61.7)	2364 (61.0)	2081 (54.7)		6807 (62.7)	2366 (51.7)	
Married or cohabiting	4836 (31.3)	1186 (29.8)	1112 (29.5)	1154 (29.8)	1384 (36.4)		3053 (28.1)	1783 (38.9)	
Divorced, separated, or widowed	1425 (9.2)	396 (9.9)	332 (8.8)	357 (9.2)	340 (8.9)		994 (9.2)	431 (9.4)	
HIV transmission route						<0.001			<0.001
Male-to-male sex contact	9878 (64.0)	2557 (64.2)	2557 (67.8)	2593 (66.9)	2171 (57.1)		7358 (67.8)	2520 (55.0)	
Heterosexual contact	4942 (32.0)	1227 (30.8)	1069 (28.3)	1147 (29.6)	1499 (39.4)		3060 (28.2)	1882 (41.1)	
IDU	130 (0.8)	42 (1.1)	30 (0.8)	31 (0.8)	27 (0.7)		91 (0.8)	39 (0.9)	
Other	484 (3.1)	157 (3.9)	115 (3.0)	104 (2.7)	108 (2.8)		345 (3.2)	139 (3.0)	
Smoking	3540 (22.9)	994 (25.0)	879 (23.3)	885 (22.8)	782 (20.6)	<0.001	2685 (24.7)	855 (18.7)	<0.001
Alcohol consumption	3517 (22.8)	772 (19.4)	807 (21.4)	954 (24.6)	984 (25.9)	<0.001	2456 (22.6)	1061 (23.2)	0.479
Glucose, mmol/L	5.16 (1.3)	5.22 (1.2)	5.16 (1.2)	5.15 (1.6)	5.11 (1.0)	0.002	5.19 (1.16)	5.09 (1.51)	<0.001
Low-density lipoprotein cholesterol, mmol/L	2.55 (0.7)	2.55 (0.7)	2.54 (0.7)	2.54 (0.7)	2.57 (0.7)	0.223	2.55 (0.69)	2.53 (0.67)	0.041
Triglycerides, mmol/L	1.45 (1.8)	1.83 (2.8)	1.42 (0.9)	1.32 (0.9)	1.22 (1.7)	<0.001	1.55 (1.86)	1.22 (1.56)	<0.001
Total cholesterol, mmol/L	4.15 (1.8)	3.67 (2.00)	4.06 (1.3)	4.25 (1.0)	4.66 (2.3)	<0.001	4.03 (1.51)	4.45 (2.21)	<0.001
Creatinine, μmol/L	73.17 (17.3)	74.74 (18.0)	73.95 (14.7)	72.96 (16.8)	70.96 (19.2)	<0.001	74.19 (16.54)	70.75 (18.82)	<0.001
WBC, 10^9/L	5.39 (1.8)	5.40 (1.9)	5.37 (1.7)	5.38 (1.7)	5.40 (1.9)	0.821	5.43 (1.81)	5.28 (1.83)	<0.001
Platelet, 10^9/L	211.69 (68.5)	209.11 (75.2)	211.76 (66.9)	211.57 (65.2)	214.46 (65.9)	0.008	211.93 (69.77)	211.12 (65.44)	0.499
HBV infection	10346 (67.0)	2329 (58.5)	2535 (67.2)	2808 (72.5)	2674 (70.3)	<0.001	7044 (64.9)	3302 (72.1)	<0.001
HCV infection	190 (1.2)	53 (1.3)	41 (1.1)	44 (1.1)	52 (1.4)	0.608	122 (1.1)	68 (1.5)	0.076
HIV RNA, copies/mL						<0.001			<0.001
<5000	1185 (7.7)	299 (7.5)	231 (6.1)	289 (7.5)	366 (9.6)		814 (7.5)	371 (8.1)	
5000-99999	5096 (33.0)	951 (23.9)	1216 (32.2)	1436 (37.1)	1493 (39.2)		3477 (32.0)	1619 (35.3)	
>=100000	9153 (59.3)	2733 (68.6)	2324 (61.6)	2150 (55.5)	1946 (51.1)		6563 (60.5)	2590 (56.6)	
CD4 count, cells/μL						<0.001			<0.001
<200	6227 (40.3)	2002 (50.3)	1540 (40.8)	1342 (34.6)	1343 (35.3)		4399 (40.5)	1828 (39.9)	
200-349	6247 (40.5)	1421 (35.7)	1504 (39.9)	1679 (43.3)	1643 (43.2)		4295 (39.6)	1952 (42.6)	
350-499	2960 (19.2)	560 (14.1)	727 (19.3)	854 (22.0)	819 (21.5)		2160 (19.9)	800 (17.5)	
CD8 count, cells/μL						<0.001	937.53 (546.22)	839.95 (480.83)	<0.001
<500	2731 (17.7)	708 (17.8)	597 (15.8)	627 (16.2)	799 (21.0)		1806 (16.6)	925 (20.2)	
500-999	7520 (48.7)	1749 (43.9)	1785 (47.3)	1972 (50.9)	2014 (52.9)		5109 (47.1)	2411 (52.6)	
>=1000	5183 (33.6)	1526 (38.3)	1389 (36.8)	1276 (32.9)	992 (26.1)		3939 (36.3)	1244 (27.2)	
CD4/CD8 ratio						<0.001	228.69 (131.18)	229.36 (122.58)	<0.001
<0.1	2419 (15.7)	967 (24.3)	582 (15.4)	424 (10.9)	446 (11.7)		1845 (17.0)	574 (12.5)	
0.1-0.39	9542 (61.8)	2510 (63.0)	2446 (64.9)	2468 (63.7)	2118 (55.7)		6765 (62.3)	2777 (60.6)	
0.4-0.79	3210 (20.8)	475 (11.9)	690 (18.3)	922 (23.8)	1123 (29.5)		2087 (19.2)	1123 (24.5)	
>=0.8	263 (1.7)	31 (0.8)	53 (1.4)	61 (1.6)	118 (3.1)		157 (1.4)	106 (2.3)	
Opportunistic infections	898 (5.8)	276 (6.9)	179 (4.7)	177 (4.6)	266 (7.0)	<0.001	606 (5.6)	292 (6.4)	0.06
Time interval, months						<0.001			<0.001
<1	8102 (52.5)	2191 (55.0)	1965 (52.1)	1979 (51.1)	1967 (51.7)		5859 (54.0)	2243 (49.0)	
1-5	4080 (26.4)	961 (24.1)	997 (26.4)	1038 (26.8)	1084 (28.5)		2772 (25.5)	1308 (28.6)	
>=6	3252 (21.1)	831 (20.9)	809 (21.5)	858 (22.1)	754 (19.8)		2223 (20.5)	1029 (22.5)	
Recent ART treatment regimen						<0.001			<0.001
3TC+TDF+EFV/NVP	7434 (48.2)	1688 (42.4)	1828 (48.5)	1981 (51.1)	1937 (50.9)		5190 (47.8)	2244 (49.0)	
DTG-containing	4310 (27.9)	1312 (32.9)	1071 (28.4)	1008 (26.0)	919 (24.2)		3280 (30.2)	1030 (22.5)	
3TC/AZT+EFV/NVP/LPV/r	1437 (9.3)	383 (9.6)	346 (9.2)	337 (8.7)	371 (9.8)		808 (7.4)	629 (13.7)	
3TC+LPV/r+TDF/AZT/D4T	1125 (7.3)	288 (7.2)	255 (6.8)	270 (7.0)	312 (8.2)		800 (7.4)	325 (7.1)	
EVG/c/FTC/TAF	677 (4.4)	180 (4.5)	169 (4.5)	167 (4.3)	161 (4.2)		468 (4.3)	209 (4.6)	
Other	451 (2.9)	132 (3.3)	102 (2.7)	112 (2.9)	105 (2.8)		308 (2.8)	143 (3.1)	

^a^Continuous variables are expressed as mean (SD). Categorical variables are expressed as frequency (percentage).

HDL, High-density lipoprotein cholesterol; BMI, body-mass index; IDU, injection drug use; ULN, upper limit of normal (45U/L); WBC, white blood cells; ART, antiretroviral therapy; HBV, hepatitis B virus; HCV, hepatitis C virus; HIV, human immunodeficiency virus; Time interval, the time between the diagnosis of HIV and the initiation of ART; 3TC, lamivudine; TDF, tenofovir disoproxil fumarate; EFV, efavirenz; NVP, nevirapine; DTG, Dolutegravir; AZT, zidovudine; LPVr, lopinavir/ritonavir; D4T, stavudine; EVG, Elvitegravir; FTC, Emtricitabine; TAF, Tenofovir Alafenamide.

### 3.2 Association of baseline HDL-C levels and their trajectories with CD4+ T-cell count recovery

**Figure 1:**
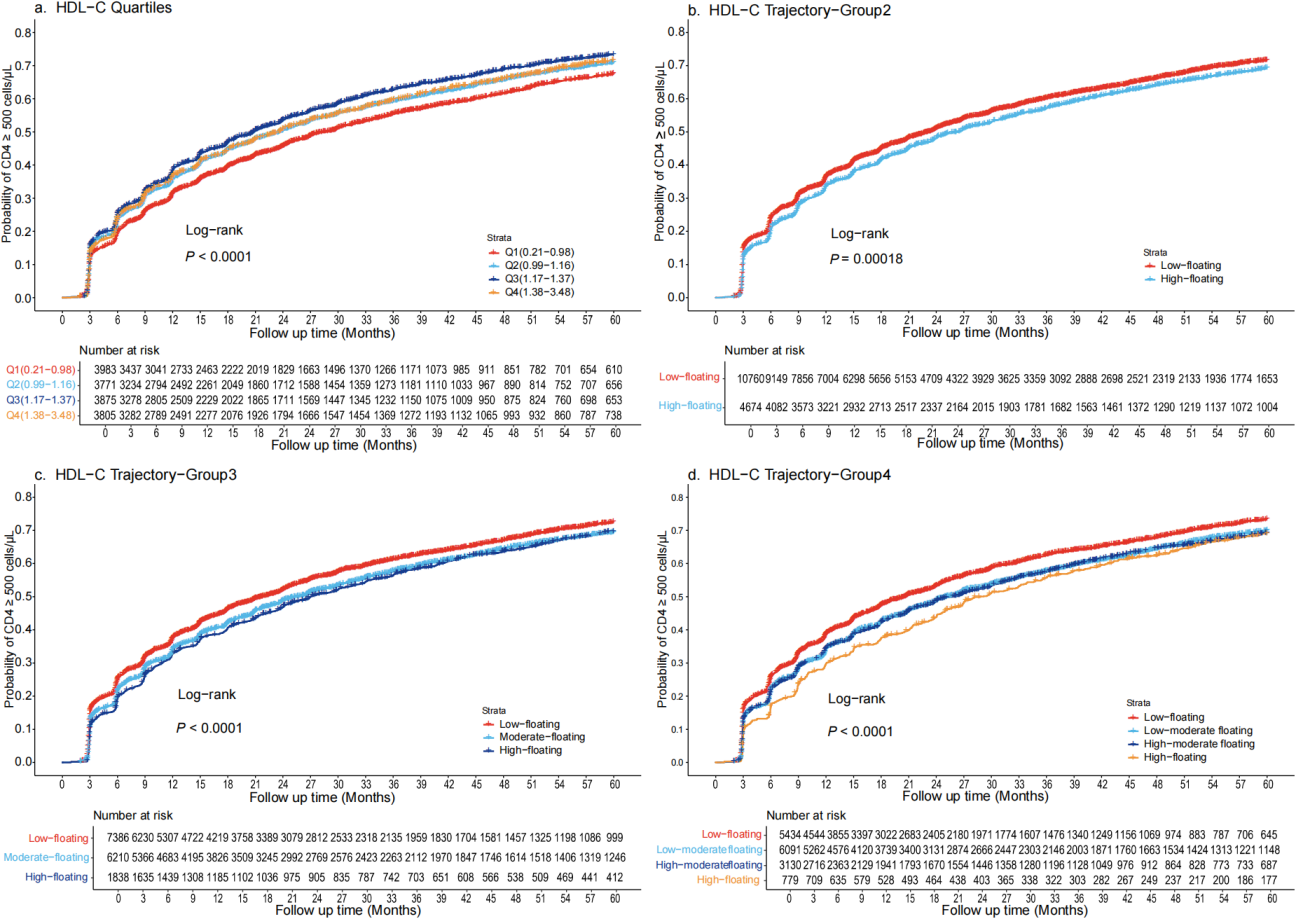
Kaplan-Meier survival curves for the cumulative probability of CD4+ T-cell count recovery, stratified by quartiles and trajectory groups in PLWH **(a)** Quartiles are defined as Q1 (0.21-0.98 mmol/L), Q2(0.99-1.17 mmol/L), Q3(1.18-1.37 mmol/L), and Q4(1.38-3.48 mmol/L). CD4+ T-cell recovery was highest in Q3 and lowest in Q1, with significant divergence in survival curves (log-rank p < 0.001). **(b)** Trajectory are categorized as Low-floating (69.7%) and High-floating (30.2%). CD4+ T-cell recovery was notably higher in the Low-floating group (log-rank p < 0.00018). **(c)** Trajectory are categorized as Low-floating (47.8%), Moderate-floating (40.2%), High-floating (11.9%). The Low-floating group had the highest recovery rate (log-rank p < 0.0001). **(d)** Trajectory are categorized as Low-floating (35.2%), Low-moderate floating (39.4%), High-moderate floating (20.2%), High-floating (5%). The Low-floating group exhibited the highest CD4+ T-cell recovery rate (log-rank p < 0.0001). HDL-C, High-density lipoprotein cholesterol; PLWH, people living with HIV.

During a total follow-up of 32,525.31 person-years, 9,609 participants achieved CD4+ T-cell recovery to 500 cells/μL or higher. Kaplan–Meier survival curves showed that 73.3% of individuals in the Q3 of HDL-C group achieved CD4+ T-cell counts ≥500 cells/µL at five years, while individuals in the Q1, Q2, and Q4 groups reached this level at probabilities of 67.5%, 71%, and 71.5%, respectively (**Figure 1a**). In an unadjusted Cox regression analysis, Q3 had the highest probability for CD4+ T-cell recovery, consistent with the Kaplan-Meier curves results. These associations persisted in model 2, although the hazard ratios (HRs) were slightly reduced compared to model 1 (p < 0.001). After full covariate adjustment, compared to Q1, the HRs (95% confidence intervals [CIs]) for Q2, Q3, and Q4 were 0.94 (95% CI: 0.88-0.99), 0.92 (95% CI: 0.87-0.98), and 0.85 (95% CI: 0.80-0.91), respectively (p < 0.001) (**Table 2**). Addition of HDL-C to the model that included all significant factors for CD4+ T-cell recovery improved the predictive risk as showed by the metrics of reclassification (NRI=0.31, 95% CI: 0.27-0.34, p < 0.05) (**Supplementary Table S4**).

**Table 2:** Association between HDL-C and achieving CD4 counts above 500 cells/μL within 5 years.

Measure/Model	Quartiles of HDL-C				Trajectory−2Groups of HDL-C		*P*-trend
	Quartile 1	Quartile 2	Quartile 3	Quartile 4	Low-floating	High-floating	
Median (mmol/L)	0.87	1.08	1.26	1.54	1.08	1.42	
No. of cases/Person-months	98680.73/390303.7	100479.93/390303.7	89576.20/390303.7	99567.20/390303.7	258293.8/390303.7	132009.9/390303.7	
Model 1, HR (95% CI)	1.00	1.13 (1.07-1.20)	1.22 (1.15-1.29)	1.14 (1.08-1.21)	1.00	0.85(0.82-0.89)	0.001
Model 2, HR (95% CI)	1.00	1.10 (1.03-1.16)	1.19 (1.13-1.26)	1.15 (1.09-1.22)	1.00	0.88(0.85-0.93)	<0.001
Model 3, HR (95% CI)	1.00	0.94 (0.88-0.99)	0.92 (0.87-0.98)	0.85 (0.80-0.91)	1.00	0.86(0.82-0.90)	<0.001

HDL, High-density lipoprotein cholesterol; HR, hazard ratio; CI, confidence interval; ART, antiretroviral therapy.

Model 1: an unadjusted model.

Model 2: adjusted for sex, age.

Model 3: adjusted for sex, age, body mass index, smoking, alcohol consumption, white blood cells, HIV transmission route, CD8 count, CD4 count, CD4/CD8 ratio, HIV RNA, HBV, HCV, ART treatment regimen, opportunistic infections, the time interval between HIV diagnosis and ART initiation.

Compared to the traditional approach of using static baseline HDL-C quartiles, the dynamic HDL-C group trajectory categorization demonstrated notable differences in its predictive power for CD4+ T-cell recovery. Kaplan–Meier survival curves indicated that approximately 72.5% of individuals with Low-floating HDL-C levels achieved CD4+ T-cell counts ≥500 cells/µL at five years. In contrast, individuals in the High-floating group had more difficulty achieving CD4+ T-cell counts ≥ 500 cells/μL (**Figure 1b**). Similarly, when trajectory groups were divided into three and four groups, individuals with Low-floating HDL-C levels consistently exhibited higher recovery rates compared to those with moderate and high levels (**Figures 1c, d**). After full covariate adjustment in the Cox regression analysis, the HRs for the High-floating group was 0.86 (95% CI: 0.82-0.90) compared to Low-floating (p < 0.001) (**Table 2**). Regardless of whether analyzed using Kaplan-Meier survival curves or Cox regression models, CD4+ T-cell recovery consistently improved as HDL-C levels declined, a pattern that was similarly observed across the three-group and four-group trajectory models (**Supplementary Tables S5** and **S6**).

### 3.3 The detection of nonlinear relationships

**Figure 2:**
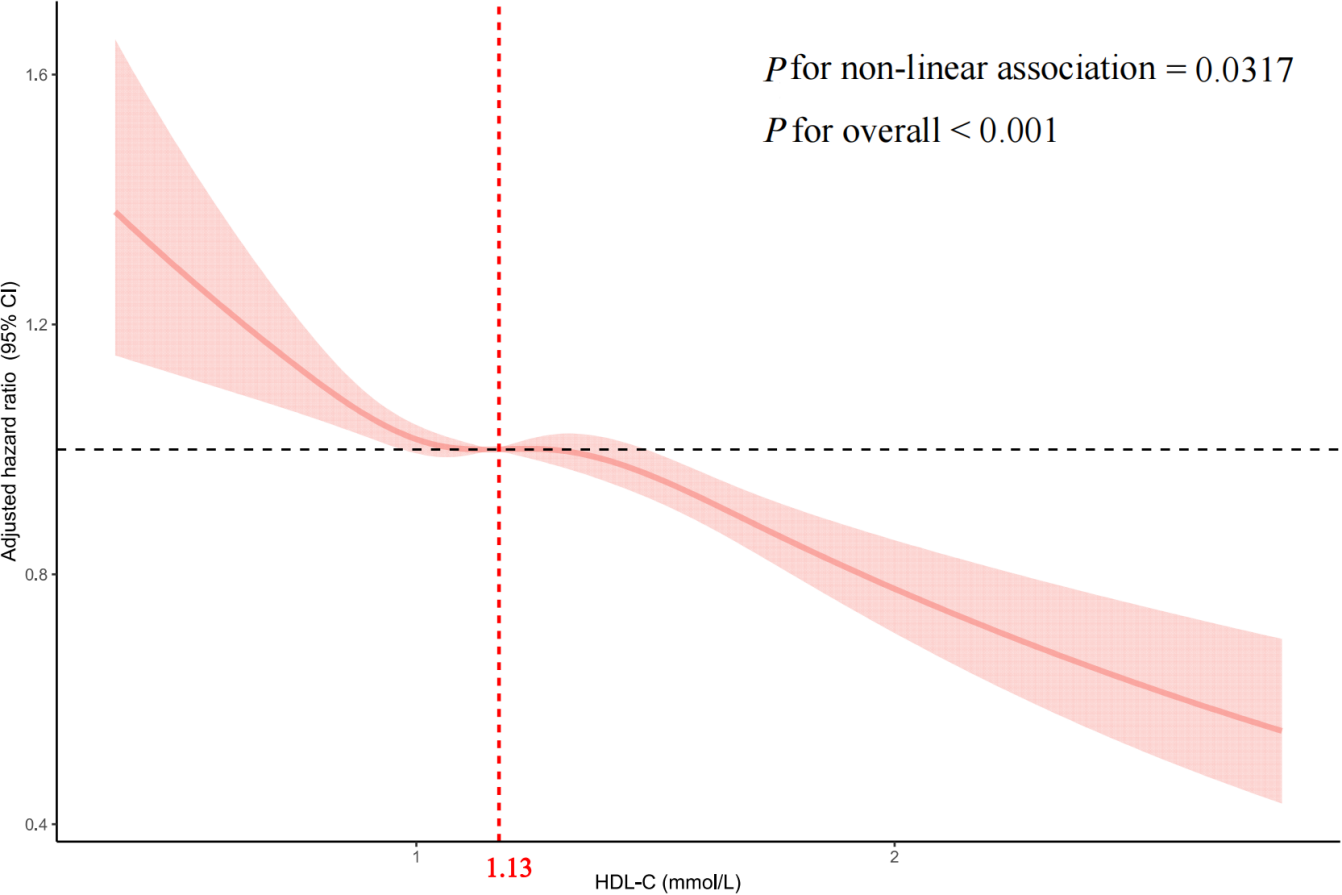
Multivariable adjusted spline analysis of HDL-C and CD4+ T-cell counts recovery to 500 cells/μL or higher in PLWH The solid line represents the adjusted HR for CD4+ T-cell count recovery, and the pink shaded area denotes the 95% CI. A nonlinear association is observed, with an inflection point at a HDL-C of 1.13 mmol/L. Covariates adjusted in the model include sex, age, body mass index, smoking, alcohol consumption, white blood cells, HIV transmission route, CD8+ T-cell count, CD4+ T-cell count, CD4/CD8 ratio, HIV RNA, HBV, HCV, ART treatment regimen, opportunistic infections, Time interval. HDL-C, High-density lipoprotein cholesterol; PLWH, people living with HIV; HR, hazard ratio; CI, confidence interval; BMI, body mass index; HBV, hepatitis B virus; HCV, hepatitis C virus; ART, antiretroviral therapy; Time interval, the time interval between HIV diagnosis and ART initiation.

Multivariate-adjusted restricted cubic spline analysis indicated a nonlinear association between HDL-C and CD4+ T-cell count recovery, with the adjusted smoothed plot revealing a consistent downward trend (p for nonlinearity = 0.0317, p for overall significance < 0.001, **Figure 2**).

We assessed the association between baseline HDL-C levels and CD4+ T-cell count recovery using two Cox proportional hazards regression models. The standard model showed an adjusted HRs of 0.79 (95% CIs: 0.73-0.85, p < 0.001). Based on a two-piecewise model, we identified an inflection point at 1.13mmol/L (p for log-likelihood ratio < 0.001; **Table 3**). After adjusting for relevant covariates, each unit increase in HDL-C below the threshold of 1.13mmol/L was associated with a 28% reduction in the likelihood of CD4+ T-cell count recovery (HR=0.72, 95% CI: 0.57-0.92, p=0.007). Concurrently, for HDL-C levels above the threshold, each unit increase was associated with 22% reduction in the likelihood of CD4+ T-cell count recovery (HR=0.78, 95% CI: 0.69-0.87, p < 0.001). In sensitivity analyses excluding individuals within six months of follow-up, HDL-C remained significantly associated with CD4+ T-cell count recovery to 500 cells/µL or higher, showing consistent patterns of recovery both below and above the 1.13mmol/L threshold (all p < 0.05; **Supplementary Table S7**). Similarly, in analyses restricted to participants enrolled within the past 10 years, HDL-C remained significantly associated with CD4+ T-cell count recovery to ≥ 500 cells/µL. A threshold effect was again observed around 1.13 mmol/L (exact value: 1.1298 mmol/L), closely aligning with the primary analysis. This nonlinear relationship persisted both below and above the threshold (all p < 0.05; **Supplementary Figure S2**, **Supplementary Table S8**).

**Table 3:** Threshold effect analysis of HDL-C on achieving CD4 counts above 500 cells/μL within 5years in people living with HIV.

Model & Subgroup	Adjusted HR (95% CI)	*P*-value
Fitting by the standard Cox proportional risk model	0.79 (0.73, 0.85)	<0.001
Fitting by the two-piecewise Cox proportional risk model		
Inflection point	1.13	
HDL-C < 1.13	0.72 (0.57, 0.92)	0.007
HDL-C >= 1.13	0.78 (0.69, 0.87)	<0.001
p for Log-likelihood ratio	<0.001	

HDL, High-density lipoprotein cholesterol; HR, hazard ratio; CI, confidence interval; ART, antiretroviral therapy.

Cox proportional hazard models were used to estimate HR and 95% CI.

Adjusted for sex, age, body mass index, smoking, alcohol consumption, white blood cells, HIV transmission route, CD8 count, CD4 count, CD4/CD8 ratio, HIV RNA, HBV, HCV, ART treatment regimen, opportunistic infections, the time interval between HIV diagnosis and ART initiation.

### 3.4 Stratified analyses

**Figure 3:**
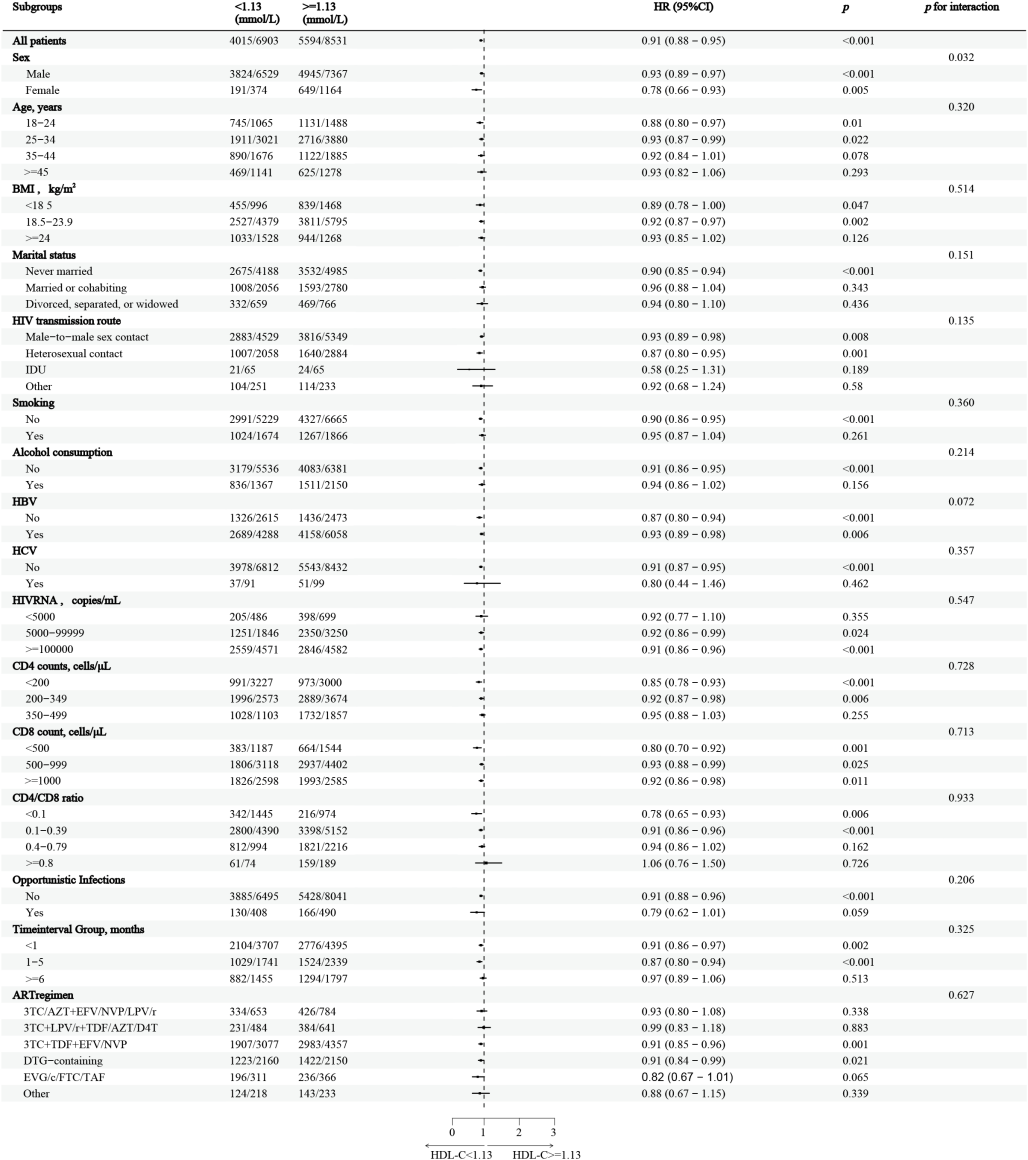
Stratified analysis of HDL-C and CD4+ T-cell counts recovery in PLWH This figure shows the HRs and 95%CIs for CD4+ T-cell count recovery associated with HDL-C levels below and above the inflection point of 1.13mmol/L, across various subgroups of people living with HIV, Adjustments were made for confounding factors in each subgroup. The vertical dashed line represents the HDL-C threshold (1.13mmol/L), while the horizontal dashed line indicates an HR of 1, indicating no increased risk. HDL-C, High-density lipoprotein cholesterol; PLWH, people living with HIV; HR, hazard ratio; CI, confidence interval.

In the stratified analysis, HDL-C levels greater than 1.13mmol/L were associated with a 9% decrease in the overall CD4+ T-cell recovery probability, compared to those with HDL-C levels below 1.13mmol/L (**Figure 3**). The reduction in recovery probability was most notable in females, individuals with a BMI less than 24kg/m^2^, those with heterosexual contact as the HIV transmission route, non-smokers, non-drinkers, and individuals without HBV or HCV infections. Additionally, individuals with HIV RNA levels above 5,000 copies/mL, CD4+ T-cell counts below 350 cells/μL, CD8 counts below 500 cells/μL, and those without opportunistic infections demonstrated significant reductions in CD4+ T-cell recovery probability. Participants who initiated ART within six months of diagnosis, as well as those on 3TC+TDF+EFV/NVP and DTG-containing regimens, also displayed reduced recovery probability. Furthermore, a significant interaction between gender and HDL-C levels was observed (p = 0.032).

## 4 Discussion

This study is the first systematic investigation into the role of HDL-C in immune reconstitution among PLWH, highlighting its potential as a predictor of immune recovery. By analyzing baseline HDL-C levels and employing a restricted cubic spline model, we identified a significant nonlinear association between HDL-C and CD4+ T-cell recovery, with 1.13 mmol/L emerging as a key inflection point. This threshold effect offers new insights into the complex role of HDL-C in immune regulation. Additionally, the application of GBTM allowed us to explore the influence of dynamic HDL-C changes on immune recovery. We identified distinct subgroups with similar HDL-C trajectories, revealing the relationship between HDL-C dynamics and long-term immune recovery patterns. This innovative approach transcends traditional cross-sectional analyses by enabling an examination of the sustained effects of HDL-C changes over time on immune reconstitution. Collectively, these findings fill an important gap in the understanding oh HDL-C’s role in HIV-relaved immune recovery and provide valuable insights for the development of future clinical interventions and personalized treatment strategies.

Our study suggests that HDL-C levels are closely associated with immune reconstitution in PLWH, indicating that HDL-C may serve as a potential predictive biomarker for CD4+ T-cell recovery. Beyond its classical role in cholesterol transport, HDL-C is increasingly recognized for its immunomodulatory functions. Specifically, HDL-C has been implicated in T-cell activation and survival by facilitating cholesterol efflux and stabilizing lipid rafts, both of which are critical for CD4+ T-cell functionality (29). In the setting of chronic HIV infection, however, HDL-C may become oxidized or dysfunctional, thereby losing its anti-inflammatory properties and potentially contributing to persistent immune activation. This notion is supported by previous study reporting elevated IL-6 and sCD163 levels in ART-treated individuals with altered HDL-C profiles, indicating ongoing inflammation despite virologic suppression (30). Moreover, the observed nonlinear relationship—with a threshold at 1.13 mmol/L—further highlights the complex and context-dependent role of HDL-C in immune regulation. In individuals with HDL-C levels below this threshold, elevations may reflect early metabolic disturbance or heightened immune stress, during which HDL-C particles are more likely to be quantitatively insufficient and functionally impaired. In this state, increasing HDL-C levels may fail to confer immunologic benefit and instead contribute to immune dysregulation. Above the threshold, although HDL-C levels are higher, a proportion of particles may still be dysfunctional due to chronic inflammation (31, 32). However, the presence of residual functional HDL-C—capable of maintaining antioxidative and cholesterol efflux functions—may attenuate the adverse immunological effects of further increases. This may explain why the negative association between HDL-C and immune recovery appears to weaken at higher concentrations. Nevertheless, even at these elevated levels, dysfunctional HDL-C may continue to sustain immune activation and impair CD4+ T-cell homeostasis, thereby limiting immune reconstitution.

Stratified analyses further revealed that the effect of HDL-C on immune recovery varies across clinical subgroups, suggesting potential biological interactions with sex, BMI, and smoking status. In women, estrogen modulates both HDL-C functionality and gut microbial composition. The decline in estrogen during menopause has been associated with increased microbial translocation and immune activation, which may in turn promote the glycation and oxidation of HDL-C particles. These dysfunctional HDL-C particles may lose their anti-inflammatory capacity and instead exacerbate immune activation, thereby impairing CD4+ T-cell recovery and limiting immune reconstitution (33, 34). Among individuals with lower BMI, disturbances in HDL-C metabolism—such as reduced synthesis of apolipoprotein A-I and dysregulation of lipid-processing like lipoprotein lipase—may compromise the immunomodulatory function of HDL-C (35). These changes can promote a pro-inflammatory phenotype through the activation of TLR2/4–NF-kB signaling pathways, potentially limiting immune recovery despite effective viral suppression (36). In non-smokers, the absence of nicotine-induced miRNA-141 expression may preserve HDL-C’s antioxidative and cholesterol transport functions. While these functions are generally protective, under conditions of chronic HIV infection, they may paradoxically enhance TLR-mediated immune activation and sustain low-grade inflammation. This persistent immune activation, despite effective ART, could ultimately impair CD4+ T-cell recovery and contribute to suboptimal immune reconstitution outcomes in this subgroup (37).

These findings underscore the necessity for further inquiry into the mechanisms by which HDL-C impacts immune reconstitution, particularly its interaction with inflammation and immune system dysfunction. The stronger associations observed in women, lean individuals, and non-smokers highlight that HDL-C’s immunological effects may vary depending on host characteristics. Clinically, integrating HDL-C monitoring into HIV management could help identify individuals at higher risk for suboptimal immune recovery, thereby improving risk stratification and informing early interventions such as personalized treatment strategies. However, the generalizability of HDL-C guided predictions across diverse populations, including those with differing comorbidities, hormonal status, or nutritional profiles, warrants further investigation. Prospective research targeting HDL-C and associated inflammatory pathways may provide new therapeutic to enhance immune recovery in PLWH.

GBTM is a valuable tool to analyze longitudinal disease histories by identifying distinct clusters (38). In our study, we utilized this approach to identify distinct HDL-C trajectory patterns among PLWH. It transcends conventional baseline analyses, which are limited to a single time point and overlook individual variability. By tracing the longitudinal HDL-C trajectories and stratifying PLWH into distinct cohorts, our methodology offers a precise depiction of the dynamic evolution of HDL-C levels and highlights the heterogeneity in immune recovery processes, particularly among those with suboptimal immune reconstitution. This understanding not only offers novel insights into immune recovery, uncovering individual differences that traditional methods may ignore, but also lays a robust foundation for future clinical interventions, personalized treatment strategies, and the long-term monitoring and optimization of disease management.

A major strength of this study is the integration of both baseline and dynamic measures of HDL-C, facilitating a comprehensive analysis of its role in immune reconstitution among PLWH. By utilizing GBTM, we captured the diverse patterns of HDL-C trajectories over time, offering a more accurate representation of HDL-C’s long-term effects on immune recovery. Consequently, various patterns of HDL-C changes can capture the impact on immune function, thereby enabling the identification of effective targeted HIV infection preventive measures for different trajectory groups. Furthermore, the large sample size and extended follow-up period enhance the robustness and reliability of the findings. The elucidation of a nonlinear relationship between HDL-C and CD4+ T-cell recovery deepens our comprehension of HDL-C’s immunomodulatory role, which has potential implications for personalized treatment strategies.

This study has some limitations. As an observational study, it limits our ability to establish causality between HDL-C levels and CD4+ T-cell recovery. Additionally, the exclusion of participants with incomplete data introduces potential selection bias, which may affect the generalizability of the results. While we adjusted for multiple covariates, there may still be residual confounding factors, such as comorbidities, lipid-lowering medications use, dietary habits, or genetic variations in lipid metabolism, that were not fully accounted for. Lastly, since the study was conducted at a single center in China, the findings may not be fully generalizable to other populations with different genetic or environmental factors.

In conclusion, this study underscores the significance of HDL-C as a predictive biomarker for immune reconstitution in PLWH. The observed nonlinear association between HDL-C levels and CD4+ T-cell recovery highlights the intricate role of HDL-C in immune modulation, extending beyond its traditional function in lipid metabolism. These findings provide a basis for further exploration into the underlying mechanisms of HDL-C’s involvement in immune recovery and suggest potential opportunities for its incorporation into personalized therapeutic strategies. Routine monitoring of HDL-C levels in HIV care may improve the identification of patients at higher risk of suboptimal immune recovery, ultimately facilitating more tailored and effective clinical interventions.

## Data Availability

The raw data supporting the conclusions of this article will be made available by the authors, without undue reservation.
