# Supplementary material for: HDL-C as a novel predictor of immune reconstitution in people living with HIV: insights from a baseline-to-dynamic change cohort study in China, 2005–2022
Source: Front Immunol. 2025 May 12;16:1520615. doi: 10.3389/fimmu.2025.1520615 (PMC12104299; doi:10.3389/fimmu.2025.1520615)
Supplement: Supplementary file 1 [file DataSheet1.docx]

**Supplementary material**

**[Supplementary material](#_Toc196035409)** [1](#_Toc196035409)

**[Supplementary Figure S1.](#_Toc196035410)** [Trajectory groups of HDL-C over time in PLWH, identified using Group-Based Trajectory Modeling. 1](#_Toc196035410)

**[Supplementary Table S1.](#_Toc196035411)** [The Bayesian Information Criterion for models with varying group number groups (Two to five clusters). 2](#_Toc196035411)

**[Supplementary Table S2.](#_Toc196035412)** [Baseline characteristics by HDL-C trajectory (Three-group model). 3](#_Toc196035412)

**[Supplementary Table S3.](#_Toc196035413)** [Baseline characteristics by HDL-C trajectory (Four-group model). 6](#_Toc196035413)

**[Supplementary Table S4.](#_Toc196035414)** [Reclassification and discrimination of models incorporating HDL-C for CD4+ T-cell recovery to above 500 cells/μL in PLWH over five years. 9](#_Toc196035414)

**[Supplementary Table S5.](#_Toc196035415)** [Association between HDL-C trajectory (Three-group model) and CD4+ T-cell recovery to above 500 cells/μL within 5 years. 10](#_Toc196035415)

**[Supplementary Table S6.](#_Toc196035416)** [Association between HDL-C trajectory (Four-group model) and CD4+ T-cell recovery to above 500 cells/μL within 5 years 11](#_Toc196035416)

**[Supplementary Figure S2.](#_Toc196035417)** [Multivariable adjusted spline analysis of HDL-C and CD4+ T-cell counts recovery to 500 cells/μL or higher in PLWH (enrolled individuals between 2013 and 2022) 13](#_Toc196035417)

**[Supplementary Table S8.](#_Toc196035418)** [Sensitivity analysis of HDL-C threshold effect for CD4+ T-cell recovery to above 500 cells/μL in PLWH (enrolled individuals between 2013 and 2022). 14](#_Toc196035418)

**Supplementary Figure S1.** Trajectory groups of HDL-C over time in PLWH, identified using Group-Based Trajectory Modeling.


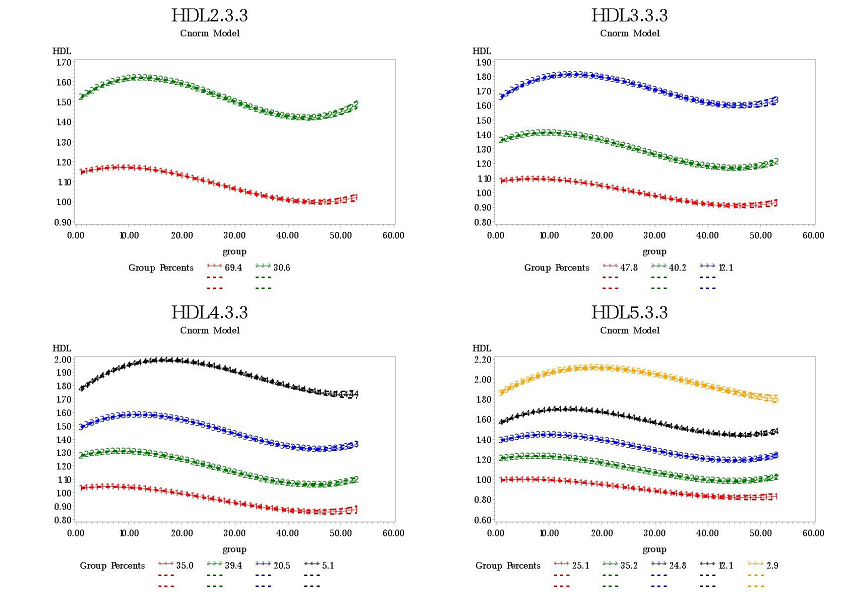


**Supplementary Table S1.** The Bayesian Information Criterion for models with varying group number groups (Two to five clusters).

| **Number of**  **Trajectory group** | **Order of the Trajectory**  **polynomials** | **BIC** | **AIC** | **No. of patients**  **classified into group** | **% of patients**  **Classified into group** | **AvePP** |
| --- | --- | --- | --- | --- | --- | --- |
| 2 | (3,3) | -8552.88 | -8514.66 | 10760 | 69.7 | 0.983 |
|  |  |  |  | 4674 | 30.2 | 0.971 |
| 3 | (3,3,3) | 19530.26 | 19587.6 | 4386 | 47.8 | 0.967 |
|  |  |  |  | 6210 | 40.2 | 0.950 |
|  |  |  |  | 1838 | 11.9 | 0.967 |
| 4 | (3,3,3,3) | 33920.88 | 33997.32 | 5434 | 35.2 | 0.950 |
|  |  |  |  | 6091 | 39.4 | 0.931 |
|  |  |  |  | 3130 | 20.2 | 0.945 |
|  |  |  |  | 779 | 5.0 | 0.966 |
| 5 | (3,3,3,3,3) | 41857.14 | 41952.69 | 3873 | 25.1 | 0.934 |
|  |  |  |  | 5497 | 35.2 | 0.905 |
|  |  |  |  | 3776 | 24.8 | 0.919 |
|  |  |  |  | 1850 | 12.1 | 0.940 |
|  |  |  |  | 438 | 2.9 | 0.965 |

According to the likelihood ratio test,the final model is a three-group model . Order: 0= zero-order (intercept), 1=linear; 2= quadratic; 3=cubic. BIC= Bayesian Information Criterion; AIC= Akaike’s Information Criterion; AvePP= Average Posterior Probability

**Supplementary Table S2.** Baseline characteristics by HDL-C trajectory (Three-group model).

| **Characteristics^a^** | **Trajectory Group3 of HDL-C** | | | | |
| --- | --- | --- | --- | --- | --- |
|  | **Total** | **Low-floating** | **Moderate-floating** | **High-floating** | ***p*-value** |
| **N** | 15434 | 7525 | 6129 | 1780 |  |
| **Sex** |  |  |  |  | <0.001 |
| male | 13896 (90.0) | 7223 (96.0) | 5442 (88.8) | 1231 (69.2) |  |
| female | 1538 (10.0) | 302 (4.0) | 687 (11.2) | 549 (30.8) |  |
| **Age, years** |  |  |  |  | <0.001 |
| 18-24 | 2553 (16.5) | 1226 (16.3) | 1074 (17.5) | 253 (14.2) |  |
| 25-34 | 6901 (44.7) | 3420 (45.4) | 2753 (44.9) | 728 (40.9) |  |
| 35-44 | 3561 (23.1) | 1769 (23.5) | 1361 (22.2) | 431 (24.2) |  |
| >=45 | 2419 (15.7) | 1110 (14.8) | 941 (15.4) | 368 (20.7) |  |
| **BMI, kg/m^2^** |  |  |  |  | <0.001 |
| <18.5 | 2464 (16.0) | 945 (12.6) | 1107 (18.1) | 412 (23.1) |  |
| 18.5-23.9 | 10174 (65.9) | 4781 (63.5) | 4192 (68.4) | 1201 (67.5) |  |
| >=24 | 2796 (18.1) | 1799 (23.9) | 830 (13.5) | 167 (9.4) |  |
| **Marital status** |  |  |  |  | <0.001 |
| Never married | 9173 (59.4) | 4758 (63.2) | 3629 (59.2) | 786 (44.2) |  |
| Married or cohabiting | 4836 (31.3) | 2052 (27.3) | 1973 (32.2) | 811 (45.6) |  |
| Divorced, separated, or widowed | 1425 (9.2) | 715 (9.5) | 527 (8.6) | 183 (10.3) |  |
| **HIV transmission route** |  |  |  |  | <0.001 |
| Male-to-male sex contact | 9878 (64.0) | 5167 (68.7) | 3879 (63.3) | 832 (46.7) |  |
| Heterosexual contact | 4942 (32.0) | 2054 (27.3) | 2017 (32.9) | 871 (48.9) |  |
| IDU | 130 (0.8) | 61 (0.8) | 58 (0.9) | 11 (0.6) |  |
| Other | 484 (3.1) | 243 (3.2) | 175 (2.9) | 66 (3.7) |  |
| **Smoking** | 3540 (22.9) | 1925 (25.6) | 1321 (21.6) | 294 (16.5) | <0.001 |
| **Alcohol consumption** | 3517 (22.8) | 1653 (22.0) | 1474 (24.0) | 390 (21.9) | 0.01 |
| **Glucose, mmol/L** | 5.16 (1.27) | 5.22 (1.17) | 5.11 (1.47) | 5.07 (0.90) | <0.001 |
| **Low-density lipoprotein cholesterol, mmol/L** | 2.55 (0.68) | 2.55 (0.69) | 2.54 (0.67) | 2.53 (0.69) | 0.326 |
| **Triglycerides, mmol/L** | 1.45 (1.78) | 1.62 (1.55) | 1.30 (1.86) | 1.23 (2.30) | <0.001 |
| **Total cholesterol, mmol/L** | 4.15 (1.76) | 3.95 (1.11) | 4.26 (1.69) | 4.64 (3.36) | <0.001 |
| **Creatinine, μmol/L** | 73.17 (17.32) | 74.50 (16.72) | 72.75 (18.30) | 68.97 (15.53) | <0.001 |
| **WBC, 10^9/L** | 5.39 (1.82) | 5.46 (1.81) | 5.34 (1.78) | 5.25 (1.95) | <0.001 |
| **Platelet, 10^9/L** | 211.69 (68.51) | 212.89 (70.18) | 210.25 (66.76) | 211.58 (67.25) | 0.082 |
| **HBV infection** | 10346 (67.0) | 4691 (62.3) | 4364 (71.2) | 1291 (72.5) | <0.001 |
| **HCV infection** | 190 (1.2) | 78 (1.0) | 91 (1.5) | 21 (1.2) | 0.06 |
| **HIV RNA, copies/mL** |  |  |  |  | <0.001 |
| <5000 | 1185 (7.7) | 581 (7.7) | 450 (7.3) | 154 (8.7) |  |
| 5000-99999 | 5096 (33.0) | 2349 (31.2) | 2118 (34.6) | 629 (35.3) |  |
| >=100000 | 9153 (59.3) | 4595 (61.1) | 3561 (58.1) | 997 (56.0) |  |
| **CD4 count, cells/μL** |  |  |  |  | <0.001 |
| <200 | 6227 (40.3) | 3108 (41.3) | 2377 (38.8) | 742 (41.7) |  |
| 200-349 | 6247 (40.5) | 2882 (38.3) | 2625 (42.8) | 740 (41.6) |  |
| 350-499 | 2960 (19.2) | 1535 (20.4) | 1127 (18.4) | 298 (16.7) |  |
| **CD8 count, cells/μL** |  |  |  |  | <0.001 |
| <500 | 2731 (17.7) | 1259 (16.7) | 1066 (17.4) | 406 (22.8) |  |
| 500-999 | 7520 (48.7) | 3441 (45.7) | 3145 (51.3) | 934 (52.5) |  |
| >=1000 | 5183 (33.6) | 2825 (37.5) | 1918 (31.3) | 440 (24.7) |  |
| **CD4/8 ratio, cells/μL** |  |  |  |  | <0.001 |
| <0.1 | 2419 (15.7) | 1361 (18.1) | 824 (13.4) | 234 (13.1) |  |
| 0.1-0.39 | 9542 (61.8) | 4709 (62.6) | 3776 (61.6) | 1057 (59.4) |  |
| 0.4-0.79 | 3210 (20.8) | 1342 (17.8) | 1433 (23.4) | 435 (24.4) |  |
| >=0.8 | 263 (1.7) | 113 (1.5) | 96 (1.6) | 54 (3.0) |  |
| **Opportunistic Infections** | 898 (5.8) | 398 (5.3) | 378 (6.2) | 122 (6.9) | 0.013 |
| **Time interval, months** |  |  |  |  | <0.001 |
| <1 | 8102 (52.5) | 4131 (54.9) | 3109 (50.7) | 862 (48.4) |  |
| 1-5 | 4080 (26.4) | 1891 (25.1) | 1676 (27.3) | 513 (28.8) |  |
| >=6 | 3252 (21.1) | 1503 (20.0) | 1344 (21.9) | 405 (22.8) |  |
| **Recent ART treatment regimen** |  |  |  |  | <0.001 |
| 3TC+TDF+EFV/NVP | 7434 (48.2) | 3526 (46.9) | 3113 (50.8) | 795 (44.7) |  |
| DTG-containing | 4310 (27.9) | 2399 (31.9) | 1533 (25.0) | 378 (21.2) |  |
| 3TC/AZT+EFV/NVP/LPV/r | 1437 (9.3) | 516 (6.9) | 607 (9.9) | 314 (17.6) |  |
| 3TC+LPV/r+TDF/AZT/D4T | 1125 (7.3) | 563 (7.5) | 423 (6.9) | 139 (7.8) |  |
| EVG/c/FTC/TAF | 677 (4.4) | 308 (4.1) | 290 (4.7) | 79 (4.4) |  |
| Other | 451 (2.9) | 213 (2.8) | 163 (2.7) | 75 (4.2) |  |

Note: ^a^Continuous variables are expressed as mean (SD). Categorical variables are expressed as frequency (percentage).

HDL:High-density lipoprotein cholesterol; BMI: body-mass index; IDU: injection drug use; ULN: upper limit of normal (45U/L); WBC: white blood cells; ART: antiretroviral therapy;HBV: hepatitis B virus; HCV: hepatitis C virus; HIV: human immunodeficiency virus; Time interval: the time between the diagnosis of HIV and the initiation of ART; 3TC: lamivudine; TDF: tenofovir disoproxil fumarate; EFV: efavirenz; NVP: nevirapine; DTG: Dolutegravir; AZT: zidovudine; LPVr: lopinavir/ritonavir; D4T: stavudine; EVG: Elvitegravir; FTC: Emtricitabine; TAF: Tenofovir Alafenamide.

**Supplementary Table S3.** Baseline characteristics by HDL-C trajectory (Four-group model).

| **Characteristics^a^** | **Trajectory Group4 of HDL-C** | | | | | |
| --- | --- | --- | --- | --- | --- | --- |
|  | **Total** | **Low-floating** | **Low-moderate floating** | **High-moderate floating** | **High-floating** | ***p*-value** |
| **N** | 15434 | 5588 | 6036 | 3056 | 754 |  |
| **Sex** |  |  |  |  |  | <0.001 |
| male | 13896 (90.0) | 5389 (96.4) | 5576 (92.4) | 2461 (80.5) | 470 (62.3) |  |
| female | 1538 (10.0) | 199 (3.6) | 460 (7.6) | 595 (19.5) | 284 (37.7) |  |
| **Age, years** |  |  |  |  |  | <0.001 |
| 18-24 | 2553 (16.5) | 888 (15.9) | 1068 (17.7) | 500 (16.4) | 97 (12.9) |  |
| 25-34 | 6901 (44.7) | 2518 (45.1) | 2751 (45.6) | 1335 (43.7) | 297 (39.4) |  |
| 35-44 | 3561 (23.1) | 1338 (23.9) | 1326 (22.0) | 719 (23.5) | 178 (23.6) |  |
| >=45 | 2419 (15.7) | 844 (15.1) | 891 (14.8) | 502 (16.4) | 182 (24.1) |  |
| **BMI, kg/m^2^** |  |  |  |  |  | <0.001 |
| <18.5 | 2464 (16.0) | 683 (12.2) | 960 (15.9) | 639 (20.9) | 182 (24.1) |  |
| 18.5-23.9 | 10174 (65.9) | 3481 (62.3) | 4116 (68.2) | 2074 (67.9) | 503 (66.7) |  |
| >=24 | 2796 (18.1) | 1424 (25.5) | 960 (15.9) | 343 (11.2) | 69 (9.2) |  |
| **Marital status** |  |  |  |  |  | <0.001 |
| Never married | 9173 (59.4) | 3555 (63.6) | 3704 (61.4) | 1616 (52.9) | 298 (39.5) |  |
| Married or cohabiting | 4836 (31.3) | 1475 (26.4) | 1821 (30.2) | 1156 (37.8) | 384 (50.9) |  |
| Divorced, separated, or widowed | 1425 (9.2) | 558 (10.0) | 511 (8.5) | 284 (9.3) | 72 (9.5) |  |
| **HIV transmission route** |  |  |  |  |  | <0.001 |
| Male-to-male sex contact | 9878 (64.0) | 3840 (68.7) | 4008 (66.4) | 1714 (56.1) | 316 (41.9) |  |
| Heterosexual contact | 4942 (32.0) | 1507 (27.0) | 1808 (30.0) | 1222 (40.0) | 405 (53.7) |  |
| IDU | 130 (0.8) | 48 (0.9) | 50 (0.8) | 30 (1.0) | 2 (0.3) |  |
| Other | 484 (3.1) | 193 (3.5) | 170 (2.8) | 90 (2.9) | 31 (4.1) |  |
| **Smoking** | 3540 (22.9) | 1462 (26.2) | 1390 (23.0) | 581 (19.0) | 107 (14.2) | <0.001 |
| **Alcohol consumption** | 3517 (22.8) | 1182 (21.2) | 1465 (24.3) | 723 (23.7) | 147 (19.5) | <0.001 |
| **Glucose, mmol/L** | 5.16 (1.27) | 5.22 (1.17) | 5.15 (1.52) | 5.08 (0.99) | 5.02 (0.77) | <0.001 |
| **Low-density lipoprotein cholesterol, mmol/L** | 2.55 (0.68) | 2.55 (0.69) | 2.56 (0.68) | 2.52 (0.67) | 2.54 (0.69) | 0.139 |
| **Triglycerides, mmol/L** | 1.45 (1.78) | 1.69 (1.70) | 1.37 (1.90) | 1.21 (0.76) | 1.29 (3.42) | <0.001 |
| **Total cholesterol, mmol/L** | 4.15 (1.76) | 3.88 (1.10) | 4.20 (1.74) | 4.44 (2.63) | 4.66 (0.97) | <0.001 |
| **Creatinine, μmol/L** | 73.17 (17.32) | 74.73 (17.55) | 73.43 (15.19) | 71.06 (20.41) | 68.04 (16.13) | <0.001 |
| **WBC, 10^9/L** | 5.39 (1.82) | 5.47 (1.83) | 5.39 (1.77) | 5.30 (1.81) | 5.14 (2.09) | <0.001 |
| **Platelet, 10^9/L** | 211.69 (68.51) | 213.23 (71.35) | 210.85 (67.22) | 211.08 (66.81) | 209.50 (63.86) | 0.191 |
| **HBV infection** | 10346 (67.0) | 3325 (59.5) | 4270 (70.7) | 2224 (72.8) | 527 (69.9) | <0.001 |
| **HCV infection** | 190 (1.2) | 53 (0.9) | 82 (1.4) | 46 (1.5) | 9 (1.2) | 0.095 |
| **HIV RNA, copies/mL** |  |  |  |  |  | <0.001 |
| <5000 | 1185 (7.7) | 455 (8.1) | 415 (6.9) | 243 (8.0) | 72 (9.5) |  |
| 5000-99999 | 5096 (33.0) | 1720 (30.8) | 2029 (33.6) | 1073 (35.1) | 274 (36.3) |  |
| >=100000 | 9153 (59.3) | 3413 (61.1) | 3592 (59.5) | 1740 (56.9) | 408 (54.1) |  |
| **CD4 count, cells/μL** |  |  |  |  |  | <0.001 |
| <200 | 6227 (40.3) | 2364 (42.3) | 2302 (38.1) | 1230 (40.2) | 331 (43.9) |  |
| 200-349 | 6247 (40.5) | 2115 (37.8) | 2524 (41.8) | 1306 (42.7) | 302 (40.1) |  |
| 350-499 | 2960 (19.2) | 1109 (19.8) | 1210 (20.0) | 520 (17.0) | 121 (16.0) |  |
| **CD8 count, cells/μL** |  |  |  |  |  | <0.001 |
| <500 | 2731 (17.7) | 914 (16.4) | 1027 (17.0) | 611 (20.0) | 179 (23.7) |  |
| 500-999 | 7520 (48.7) | 2557 (45.8) | 2946 (48.8) | 1603 (52.5) | 414 (54.9) |  |
| >=1000 | 5183 (33.6) | 2117 (37.9) | 2063 (34.2) | 842 (27.6) | 161 (21.4) |  |
| **CD4/8 ratio, cells/μL** |  |  |  |  |  | <0.001 |
| <0.1 | 2419 (15.7) | 1070 (19.1) | 854 (14.1) | 405 (13.3) | 90 (11.9) |  |
| 0.1-0.39 | 9542 (61.8) | 3499 (62.6) | 3753 (62.2) | 1846 (60.4) | 444 (58.9) |  |
| 0.4-0.79 | 3210 (20.8) | 946 (16.9) | 1326 (22.0) | 745 (24.4) | 193 (25.6) |  |
| >=0.8 | 263 (1.7) | 73 (1.3) | 103 (1.7) | 60 (2.0) | 27 (3.6) |  |
| **Opportunistic Infections** | 898 (5.8) | 282 (5.0) | 362 (6.0) | 199 (6.5) | 55 (7.3) | 0.007 |
| **Time interval, months** |  |  |  |  |  | <0.001 |
| <1 | 8102 (52.5) | 3149 (56.4) | 3077 (51.0) | 1508 (49.3) | 368 (48.8) |  |
| 1-5 | 4080 (26.4) | 1374 (24.6) | 1647 (27.3) | 845 (27.7) | 214 (28.4) |  |
| >=6 | 3252 (21.1) | 1065 (19.1) | 1312 (21.7) | 703 (23.0) | 172 (22.8) |  |
| **Recent ART treatment regimen** |  |  |  |  |  | <0.001 |
| 3TC+TDF+EFV/NVP | 7434 (48.2) | 2547 (45.6) | 3067 (50.8) | 1496 (49.0) | 324 (43.0) |  |
| DTG-containing | 4310 (27.9) | 1849 (33.1) | 1607 (26.6) | 692 (22.6) | 162 (21.5) |  |
| 3TC/AZT+EFV/NVP/LPV/r | 1437 (9.3) | 361 (6.5) | 512 (8.5) | 408 (13.4) | 156 (20.7) |  |
| 3TC+LPV/r+TDF/AZT/D4T | 1125 (7.3) | 439 (7.9) | 416 (6.9) | 222 (7.3) | 48 (6.4) |  |
| EVG/c/FTC/TAF | 677 (4.4) | 235 (4.2) | 267 (4.4) | 144 (4.7) | 31 (4.1) |  |
| Other | 451 (2.9) | 157 (2.8) | 167 (2.8) | 94 (3.1) | 33 (4.4) |  |

Note: ^a^Continuous variables are expressed as mean (SD). Categorical variables are expressed as frequency (percentage).

HDL:High-density lipoprotein cholesterol; BMI: body-mass index; IDU: injection drug use; ULN: upper limit of normal (45U/L); WBC: white blood cells; ART: antiretroviral therapy;HBV: hepatitis B virus; HCV: hepatitis C virus; HIV: human immunodeficiency virus; Time interval: the time between the diagnosis of HIV and the initiation of ART; 3TC: lamivudine; TDF: tenofovir disoproxil fumarate; EFV: efavirenz; NVP: nevirapine; DTG: Dolutegravir; AZT: zidovudine; LPVr: lopinavir/ritonavir; D4T: stavudine; EVG: Elvitegravir; FTC: Emtricitabine; TAF: Tenofovir Alafenamide.

**Supplementary Table S4.** Reclassification and discrimination of models incorporating HDL-C for CD4+ T-cell recovery to above 500 cells/μL in PLWH over five years.

| **Model** | **C statistic** | |  | **NRI (continuous)** | |  | **NRI (categorical)** | |  | **IDI** | |
| --- | --- | --- | --- | --- | --- | --- | --- | --- | --- | --- | --- |
|  | **Estimate (95% Cl)** | ***p*-value** |  | **Estimate**  **(95% Cl)** | ***p*-value** |  | **Estimate**  **(95% Cl)** | ***p*-value** |  | **Estimate (95%Cl)** | ***p*-value** |
| **Conventional model** | 0.834 (0.824, 0.844) |  |  | Reference |  |  | Reference |  |  | Reference |  |
| **Conventional model + HDL** | 0.834 (0.824, 0.844) | <0.001 |  | 0.31 (0.27, 0.34) | <0.05 |  | 0.02 (0.01, 0.03) | <0.001 |  | 0.01 (0.009, 0.01) | <0.05 |

HDL: High-density lipoprotein cholesterol;

Cl = confidence interval; NRI = net reclassification improvement; Conventional model included sex, age, body mass index, smoking, drinking, white blood cells, HIV transmission route, CD8+ T-cell count, CD4+ T-cell count, CD4/CD8 ratio, HIV RNA, HBV, HCV, ART treatment regimen, opportunistic infections, the time interval between HIV diagnosis and ART initiation

**Supplementary Table S5.** Association between HDL-C trajectory (Three-group model) and CD4+ T-cell recovery to above 500 cells/μL within 5 years.

| **Measure/Model** | **Trajectory Group3 of HDL-C** | | | ***P*-trend** |
| --- | --- | --- | --- | --- |
|  | **Low-floating** | **Moderate-floating** | **High-floating** |  |
| Median | 1.01 | 1.29 | 1.53 |  |
| No. of cases/Person-months | 171292.83/390303.7 | 164095.13/390303.7 | 54915.73/390303.7 |  |
| Model 1, HR (95% CI) | 1.00 | 0.93 (0.89-0.97) | 0.73 (0.68-0.78) | 0.001 |
| Model 2, HR (95% CI) | 1.00 | 0.93 (0.89-0.97) | 0.78 (0.73-0.84) | <0.001 |
| Model 3, HR (95% CI) | 1.00 | 0.90 (0.86-0.94) | 0.75 (0.70-0.81) | <0.001 |

HDL: High-density lipoprotein cholesterol; CI: confidence interval; ART: antiretroviral therapy

Model 1: an unadjusted model

Model 2: adjusted for sex, age

Model 3: adjusted for sex, age, body mass index, smoking, drinking, white blood cells, HIV transmission route, CD8+ T-cell count, CD4+ T-cell count, CD4/CD8 ratio, HIV RNA, HBV, HCV, ART treatment regimen, opportunistic infections, the time interval between HIV diagnosis and ART initiation

**Supplementary Table S6.** Association between HDL-C trajectory (Four-group model) and CD4+ T-cell recovery to above 500 cells/μL within 5 years

| **Measure/Model** | **Trajectory Group4 of HDL-C** | | | | ***p*-trend** |
| --- | --- | --- | --- | --- | --- |
|  | **Low-floating** | **Low-moderate floating** | **High-moderate floating** | **High-floating** |  |
| Median | 0.97 | 1.21 | 1.41 | 1.64 |  |
| No. of cases/Person-months | 122629.0/390303.7 | 155331.4/390303.7 | 88255.9/390303.7 | 24087.4/390303.7 |  |
| Model 1, HR (95% CI) | 1.00 | 0.96 (0.92-1.01) | 0.85 (0.80-0.90) | 0.68 (0.61-0.75) | 0.001 |
| Model 2, HR (95% CI) | 1.00 | 0.96 (0.92-1.00) | 0.87 (0.82-0.92) | 0.74 (0.67-0.82) | <0.001 |
| Model 3, HR (95% CI) | 1.00 | 0.90 (0.86-0.95) | 0.85 (0.80-0.90) | 0.67 (0.61-0.75) | <0.001 |

HDL: High-density lipoprotein cholesterol; CI: confidence interval; ART: antiretroviral therapy

Model 1: an unadjusted model

Model 2: adjusted for sex, age

Model 3: adjusted for sex, age, body mass index, smoking, drinking, white blood cells, HIV transmission route, CD8+ T-cell count, CD4+ T-cell count, CD4/CD8 ratio, HIV RNA, HBV, HCV, ART treatment regimen, opportunistic infections, the time interval between HIV diagnosis and ART initiation**Supplementary Table S7.** Sensitivity analysis of HDL-C threshold effect for CD4+ T-cell recovery to above 500 cells/μL in PLWH (Excluding individuals with follow-up under 6 months).

| **Model & Subgroup** | **Adjusted HR (95% CI)** | ***p*-value** |
| --- | --- | --- |
| **HDL-C achieving CD4 counts above 500 cells/μL within 5years** |  |  |
| Fitting by the standard Cox proportional risk model | 0.77 (0.70, 0.84) | <0.001 |
| Fitting by the two-piecewise Cox proportional risk model |  |  |
| Inflection point | 1.13 |  |
| HDL < 1.13 | 0.59 (0.44, 0.79) | <0.001 |
| HDL ≥ 1.13 | 0.79 (0.68, 0.91) | <0.001 |
| P for Log-likelihood ratio | <0.001 |  |

HDL: High-density lipoprotein cholesterol; HR: hazard ratio; CI: confidence interval

Cox proportional hazard models were used to estimate HR and 95% CI

Adjusted for sex, age, body mass index, smoking, drinking, white blood cells, HIV transmission route, CD8+ T-cell count, CD4+ T-cell count, CD4/CD8 ratio, HIV RNA, HBV, HCV, ART treatment regimen, opportunistic infections, the time interval between HIV diagnosis and ART initiation

**
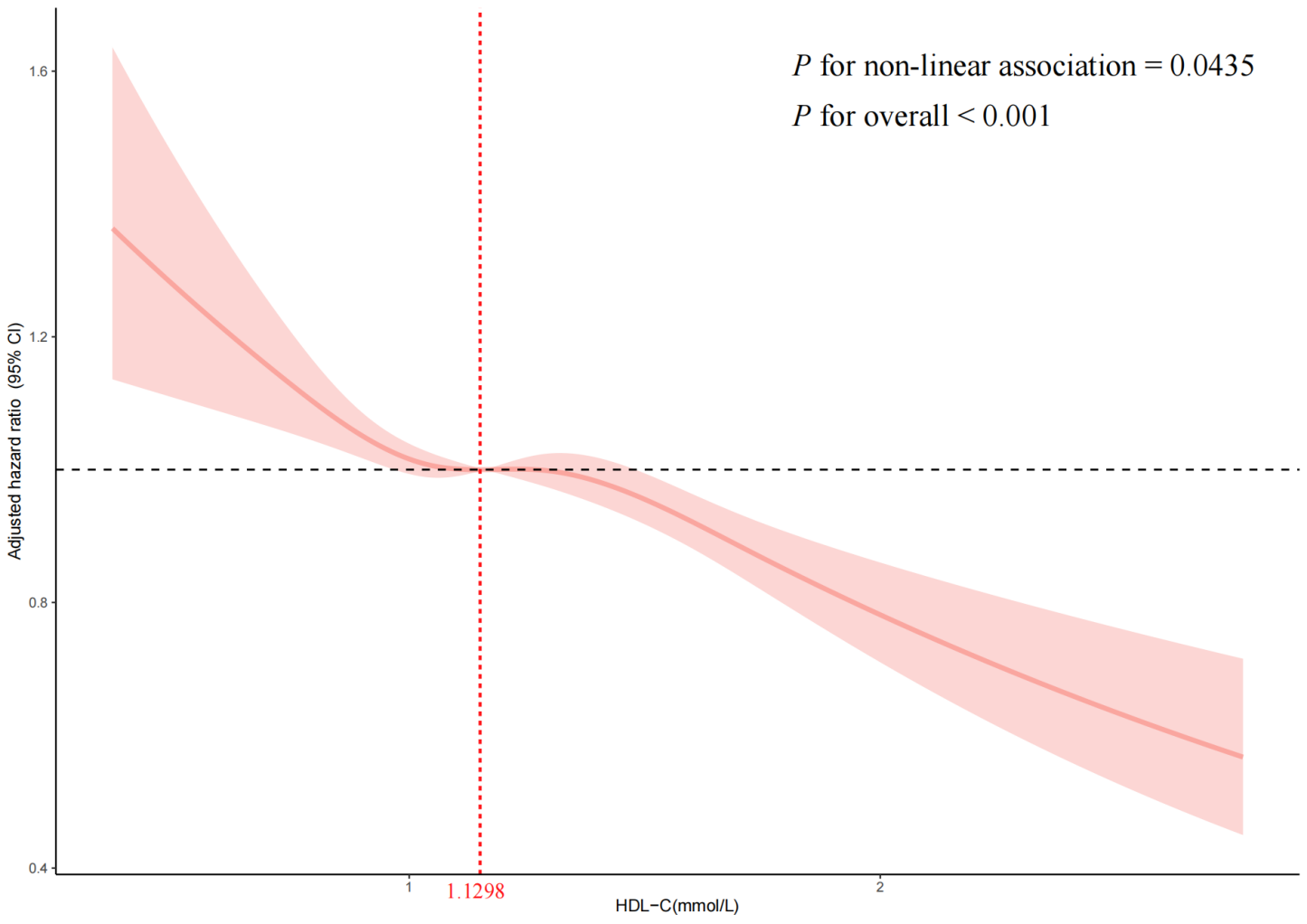
Supplementary Figure S2. Multivariable adjusted spline analysis of HDL-C and CD4+ T-cell counts recovery to 500 cells/μL or higher in PLWH (enrolled individuals between 2013 and 2022)**

The solid line represents the adjusted HR for CD4 count recovery, and the pink shaded area denotes the 95% CI. A nonlinear association is observed, with an inflection point at a HDL-C of 1.1298 mmol/L. Covariates adjusted in the model include sex, age, body mass index, smoking, alcohol consumption, white blood cells, HIV transmission route, CD8+ T-cell count, CD4+ T-cell count, CD4/CD8 ratio, HIV RNA, HBV, HCV, ART treatment regimen, opportunistic infections, Time interval.

HDL-C, High-density lipoprotein cholesterol; PLWH, people living with HIV; HR, hazard ratio; CI, confidence interval; BMI, body mass index; HBV, hepatitis B virus; HCV, hepatitis C virus; ART, antiretroviral therapy; Time interval, the time interval between HIV diagnosis and ART initiation

**Supplementary Table S8.** Sensitivity analysis of HDL-C threshold effect for CD4+ T-cell recovery to above 500 cells/μL in PLWH (enrolled individuals between 2013 and 2022).

| **Model & Subgroup** | **Adjusted HR (95% CI)** | ***P*-value** |
| --- | --- | --- |
| Fitting by the standard Cox proportional risk model | 0.79 (0.74, 0.85) | <0.001 |
| Fitting by the two-piecewise Cox proportional risk model |  |  |
| Inflection point | 1.1298 |  |
| HDL-C < 1.13 | 0.75 (0.60, 0.94) | 0.014 |
| HDL-C >= 1.13 | 0.79 (0.71, 0.88) | <0.001 |
| *P* for Log-likelihood ratio | <0.001 |  |

HDL: High-density lipoprotein cholesterol; HR: hazard ratio; CI: confidence interval; ART: antiretroviral therapy

Cox proportional hazard models were used to estimate HR and 95% CI

Adjusted for sex, age, body mass index, smoking, alcohol consumption, white blood cells, HIV transmission route, CD8+ T-cell count, CD4+ T-cell count, CD4/CD8 ratio,
